# Unexpected late-onset bleeding following thyroid surgery: case report and review of the literature

**DOI:** 10.3389/fsurg.2025.1682888

**Published:** 2025-10-20

**Authors:** Marco Marcianò, Bianca Vicari, Giuseppina Orlando, Giuseppina Melfa, Isabella Campo, Giuseppe Salamone, Gregorio Scerrino

**Affiliations:** MEPRECC “Medicina di Precisione in Area Medica, Chirurgica e Critica”, University of Palermo, Palermo, Italy

**Keywords:** hematoma, thyroidectomy, complications, antiplatelet therapy, case report

## Abstract

Postoperative hematoma is an uncommon but potentially life-threatening and unpredictable complication of thyroid surgery. We report a case of very late postoperative bleeding occurring on the eighth postoperative day (POD) in a patient undergoing right lobe thyroidectomy who had restarted antiplatelet therapy since the first POD. The patient was readmitted to the emergency department with complaints of progressive anterior neck swelling and bleeding from the wound but without respiratory distress. Under general anesthesia, cervical reexploration allowed the detection of a hematoma situated in the right thyroid bed and subhyoid region and active bleeding of the superior thyroid vascular pedicle. Although most bleeding occurs within 24 h, caution should be taken in patients who are in need of antiplatelet therapy, and close monitoring should also be advised at home after discharge.

## Introduction

Postoperative hematoma is a rare but potentially life-threatening and unpredictable complication of thyroid surgery (1–7). Its incidence is variably reported in the literature, ranging between 0.1% and 6.5%, but in major centers, it is commonly reported to be approximately 1% (1, 2, 6, 8–10). In our department, where > 150 thyroidectomies are performed per year, the incidence of postoperative bleeding in our case series is 0.7% (Scerrino et al. 2024, unpublished data).

However, identifying perioperative risk factors for the development of postoperative hemorrhage or hematoma after thyroidectomy is not really simple (7, 11, 12). Postoperative hematoma may have a multifactorial pathogenesis, including slipping of ligatures or clips, reopening of previously cauterized veins, and bleeding from residual thyroid parenchyma. Among predisposing factors, retching and bucking during recovery and perioperative increased blood pressure are considered the most significant (2, 4, 12, 13).

Although the length of stay after thyroidectomy is usually short, postoperative bleeding may be the limiting factor for early discharge from the hospital (11). While most hemorrhagic symptoms occur within 6 h after surgery, significant delays in symptoms have also been noted.

Nowadays, it is common to encounter patients on antiplatelet therapy who need surgery. According to guidelines, treatment should be discontinued 5–7 days before surgery and reintroduced 24 h after surgery (14).

Performing thyroid surgery in patients under anticoagulation/antiplatelet therapy or with coagulopathy would therefore carry a substantial but unavoidable risk of surgical wound bleeding (1).

We report a case of late postoperative bleeding occurring on the eighth postoperative day (POD) in a patient treated with cardioaspirin in secondary prevention.

## Patient information

A 58-year-old Caucasian man was referred to our department for surgical management of a toxic multinodular goiter. Surgical intervention was indicated after a routine endocrinological follow-up ultrasound revealed a suspicious nodule in the right thyroid lobe, which underwent fine-needle aspiration biopsy (FNAB) and was classified as TIR3B, according to the SIAPEC 2014 classification of thyroid nodules (15). His past medical history was significant for ischemic heart disease, with a myocardial infarction occurring approximately 5 years earlier, managed with coronary stent placement. He was maintained on chronic antiplatelet therapy with low-dose aspirin, which was discontinued 5 days prior to surgery following a cardiology consultation performed during the preoperative assessment.

Additional comorbidities included well-controlled arterial hypertension managed with an angiotensin-converting enzyme (ACE) inhibitor and hypercholesterolemia treated with a statin. No relevant family or psychiatric history was reported.

## Clinical findings

Physical examination revealed a palpable multinodular goiter. Preoperative ultrasound showed a suspicious right lobe nodule, and FNAB reported a TIR3B lesion. Although total thyroidectomy was initially indicated, a right hemithyroidectomy with isthmusectomy was performed due to type 2 signal loss detected by the nerve integrity monitor (NIM). During the operation, parathyroid glands were recognized and preserved; moreover, combined bipolar and ultrasound energy-based sealing devices and an adjunctive hemostatic patch (in particular a pad of collagen derived from cattle skin, and a crosslinked coating) were used to ensure an accurate hemostasis. The patient had restarted antiplatelet therapy on the first POD. The immediate postoperative course was uneventful. Specifically, the patient's blood pressure was monitored hourly during the first 4 h after surgery; subsequently, checks were carried out every 8 h for the entire duration of the hospital stay. No hypertensive peaks were observed during any of these checks. However, the patient resumed his normal ACE-inhibitor therapy on the first POD and was discharged on the second POD (as usual in our unit) in good general condition. At discharge, serum calcium was 9 mg/dL [normal range (nr): 8.6–10.2], and serum phosphorus was 3.4 mg/dL (nr: 2.5–4.5). At home, he continued his antiplatelet therapy as prescribed by his referring cardiologist.

## Diagnostic assessment

**Figure 1:**
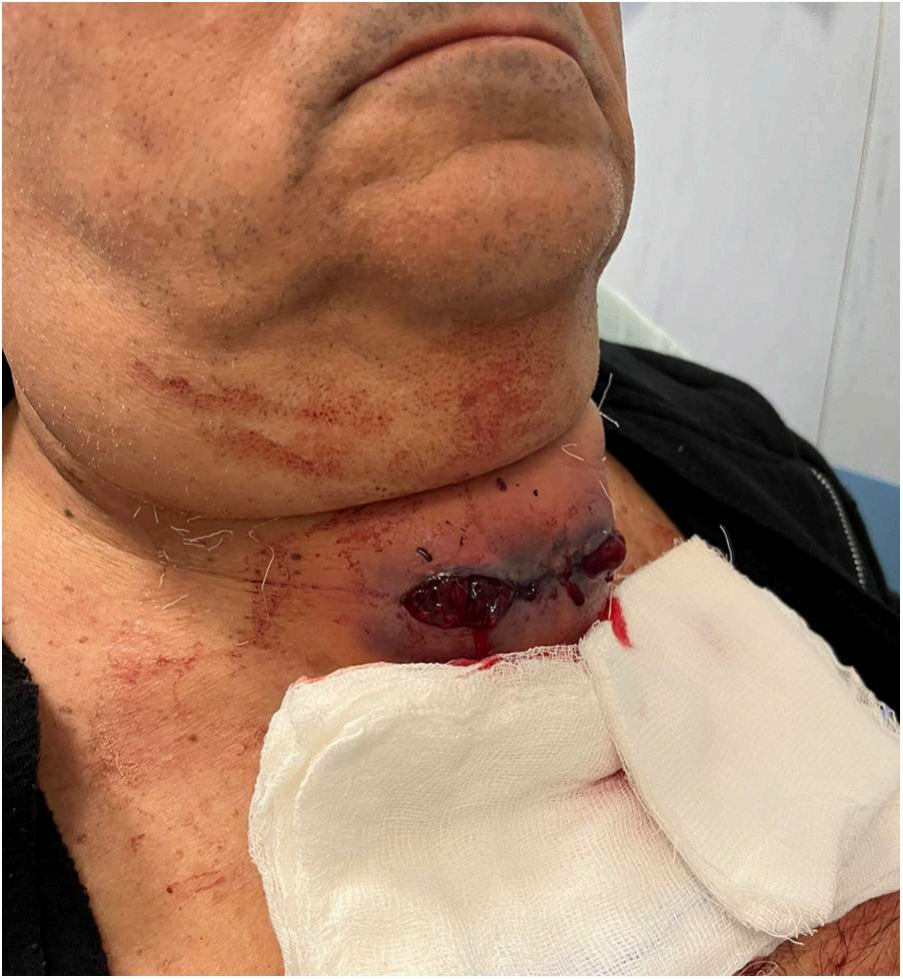
Clinical appearance of the postoperative cervical wound demonstrating significant tumefaction with extensive hemorrhagic infiltration of the surrounding soft tissues and multiple blood clots within the surgical bed. The hematoma leads to visible neck bulging and compressive symptoms, requiring prompt evaluation and intervention.

The following 5 days postoperative discharge were characterized by regular wellness; however, on the eighth postoperative day, he was readmitted as an emergency with complaints of progressive anterior neck swelling and bleeding from the wound without respiratory distress (Figure 1). Vigorous coughing, sneezing, or other acute reasons for hemorrhage were not reported by the patient.

## Therapeutic intervention

After approximately 2 h of continuous monitoring, during which active bleeding from the wound persisted, progressive cervical edema was observed. Vital signs showed clinical deterioration (SpO_2_ 85%, non-invasive blood pressure (NIBP) 150/90 mmHg, HR 130 bpm), and the patient was taken to the operating room for emergency surgical intervention on the same day.

Under general anesthesia, cervical exploration was performed, allowing the detection of a hematoma situated in the right thyroid bed and subhyoid region and an active bleeding of the superior thyroid artery that was arrested by selective ligation. Additional hemostatic control was performed by placing one vial of hemostatic collagen and human thrombin gel (floseal) in the right thyroid bed.

Antiplatelet therapy was restarted 24 h after surgery, and the patient was discharged 2 days after surgery in good condition, without symptoms and without any sign of further bleeding.

## Follow-up and outcomes

Histopathological examination of the right thyroid lobe revealed a multifocal, encapsulated classical variant of papillary thyroid carcinoma (WHO 2022 classification), extensively involving almost the entire lobe and the isthmus, with surgical resection margins free of tumor infiltration. In the 20th POD, the complete recovery of right vocal fold motility was observed. In accordance with current clinical guidelines, the patient underwent a complete thyroidectomy 3 months later. Upon readmission, a left lobectomy was performed under ordinary inpatient care; a drain was placed in the thyroid bed and removed on the first postoperative day, and the patient was discharged on the third postoperative day in good condition. Histopathological examination of the left thyroid lobe demonstrated a 4 mm focus of classical variant papillary thyroid carcinoma (WHO 2022), located in the upper third of the lobe, with uninvolved surgical margins. Timeline of the surgical event was summarized in Table 1.

**Table 1:** Timeline.

Date/event	Details
Day-5	Discontinued cardioaspirin
Day 0	Right hemithyroidectomy with isthmusectomy performed
Day 1	Cardioaspirin restarted
Day 2	Discharged in stable condition
Day 8	Presented with neck swelling and wound bleeding
Day 8	Emergency reoperation: active bleeding from the superior thyroid artery
Day 10	Discharged without complications
3 months later	Completion thyroidectomy performed after carcinoma diagnosis

## Discussion

Hematoma following thyroid surgery is a rare but potentially life-threatening complication, with a reported rate of 0.1%–4.7% (3, 6, 14).

Meticulous hemostatic techniques are necessary to prevent this complication; coagulation devices and hemostatic agents are currently the most deployed resources among surgeons (2, 16). Recent meta-analytic evidence indicates that energy-based devices outperform conventional clamp-and-tie ligatures in total thyroidectomy, providing more efficient hemostasis in highly vascularized areas and reducing the incidence of transient recurrent laryngeal nerve (RLN) palsy, hypocalcemia, and other postoperative complications, including cervical hematoma (17). In line with these findings, a comprehensive review highlighted that postoperative hematoma, which occurs in approximately 0.1%–1.1% of thyroidectomy cases—mostly within the first 6 h—remains a major surgical concern, and that the introduction of advanced bipolar and ultrasonic energy devices has significantly reduced operative time without increasing costs or complication rates (18, 19).

In addition, topical hemostatic agents have been shown to effectively control bleeding during thyroidectomy. A recent study involving 84 patients demonstrated that both oxidized regenerated cellulose (Oxitamp®) and fibrin glue (Tisseel®) provide reliable hemostasis, with fibrin glue exhibiting a more favorable ultrasound profile, thereby minimizing the risk of residual material being misinterpreted as thyroid tissue or tumor recurrence (20). Another randomized study of 155 patients undergoing total thyroidectomy compared Floseal with conventional hemostatic techniques and Tabotamp, reporting that Floseal significantly reduced operative time, enabled earlier drain removal, and shortened postoperative hospital stay, supporting its use as an effective adjunct in thyroid surgery (21). Furthermore, a large multicenter randomized trial of 206 patients demonstrated that the combination of Floseal and an ultrasound scalpel significantly reduced 24 h drain output and operative time compared with standard thyroidectomy, supporting its role as a complementary hemostatic strategy (22).

An increasing number of ambulatory and 1-day surgery thyroidectomies have been performed in the last several years (2). Postoperative bleeding is one of the limiting factors that preclude early discharge (9).

It is not simple to predict the risk of developing a hematoma after thyroid surgery among patients (2). However, some factors that can be associated with a higher risk of bleeding seem to be as follows: male gender, hyperthyroidism, intrathoracic goiters, and reoperative surgery (2, 7, 8, 11, 23, 24). Recent studies have identified key risk factors for postoperative cervical hematoma: in a multicenter cohort of 8,839 patients, male sex, older age, higher body mass index, unilateral lateral neck dissection, drain placement, and shorter operative times were associated with hematoma, which occurred in 3.15% of cases, mostly within 6 h (25). UK registry data similarly highlighted male sex, older age, redo surgery, retrosternal goiter, and total thyroidectomy as significant predictors, although overall bleeding remains difficult to predict (26). In a single-center analysis of 5,900 thyroidectomies, cervical hematoma occurred in 1.1% of patients, with the inferior thyroid artery and superior thyroid vessels as the most common bleeding sources; older age, concurrent lymph node dissection, and chronic lymphocytic thyroiditis were significant contributors (27).

However, one of the most likely causes of bleeding seems to be due to postsurgical hypertension. Therefore, very close monitoring of pressure during the first 24 h after surgery and prompt treatment of all manifestations of hypertension with appropriate drugs are recommended (2). It has also been associated with the administration of anticoagulants or coagulation alterations, such as hemophilia, von Willebrand's disease, and chronic renal failure (2).

The perioperative management of low-dose aspirin (cardioaspirin) in patients undergoing thyroid surgery requires a careful balance between minimizing thromboembolic events and reducing hemorrhagic complications. Current guidelines, such as those from the American College of Cardiology (ACC) and American Heart Association (AHA), recommend continuing low-dose aspirin therapy in patients with coronary artery disease undergoing non-cardiac surgery when the bleeding risk is acceptable to the surgical team. Specifically, for patients with bare-metal stents, it is advised to continue aspirin therapy through the perioperative period unless the surgical bleeding risk is considered too high.

In procedures with elevated bleeding risk, some authors suggest withholding aspirin 5–10 days before surgery (28, 29). Clinical evidence in thyroid surgery suggests that continuing aspirin does not significantly increase the risk of intraoperative or postoperative bleeding, although some studies report no difference in bleeding complications between patients who continue or withhold therapy (30). Therefore, the decision to continue or suspend low-dose aspirin should be individualized, considering the patient's cardiovascular risk, the type of surgery, and collaboration between cardiology and surgical teams is recommended to optimize perioperative management and ensure patient safety (28–30).

However, the decision to continue or discontinue low-dose aspirin should be individualized, considering the patient's cardiovascular risk profile and the specific surgical procedure. In patients undergoing thyroid surgery, particularly those with coronary artery disease or recent stent placement, continuing low-dose aspirin therapy may be beneficial in preventing thromboembolic events without significantly increasing the risk of bleeding. Collaboration between cardiology and surgical teams is essential to optimize perioperative management and ensure patient safety.

Although many studies only focus on the risk factors of bleeding after thyroid surgery, it is important to also pay attention to the most frequent sources of bleeding, so that more attention can be paid to them during surgery.

**Figure 2:**
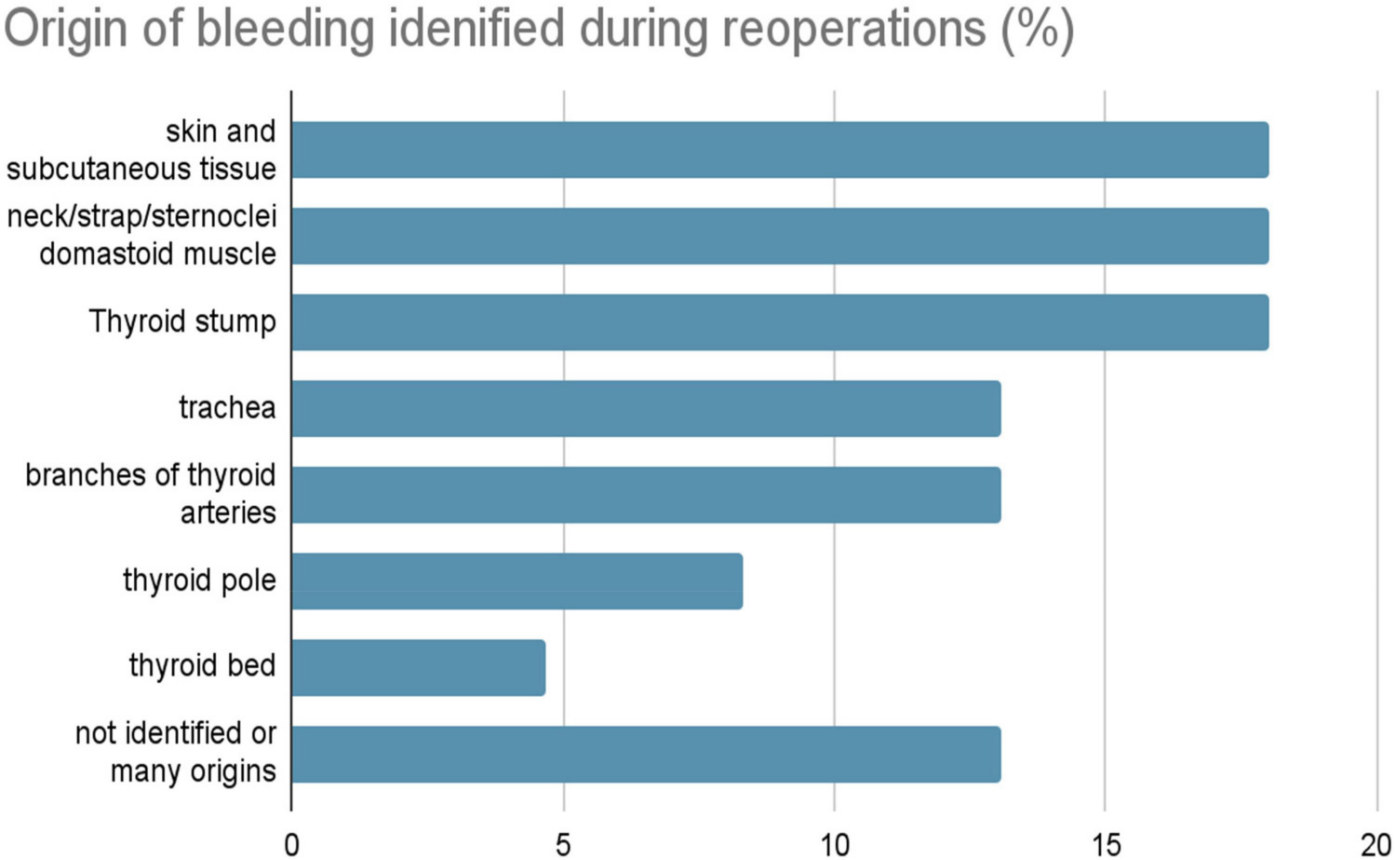
Bar chart illustrating the distribution of bleeding sources identified during reoperations for postoperative cervical hematoma following thyroid surgery. The most frequently identified bleeding origins were the skin and subcutaneous tissue, strap and sternocleidomastoid muscles, and the thyroid stump (each accounting for approximately 18%–19% of cases). Bleeding from branches of the thyroid arteries and the trachea was observed in approximately 13%–14% of cases, while the thyroid pole represented approximately 8% and the thyroid bed approximately 5%. In nearly 15% of cases, the bleeding source could not be precisely identified or was attributed to multiple origins.

According to a study that analyzed multiple cases of reoperation due to bleeding after thyroid surgery, although it is not always possible to identify a precise or single source, the most common are listed in Figure 2. Particularly in our case, the bleeding resulted from a leakage of the superior thyroid artery (thus finding ourselves in 13.1% of all cases) (31).

**Figure 3:**
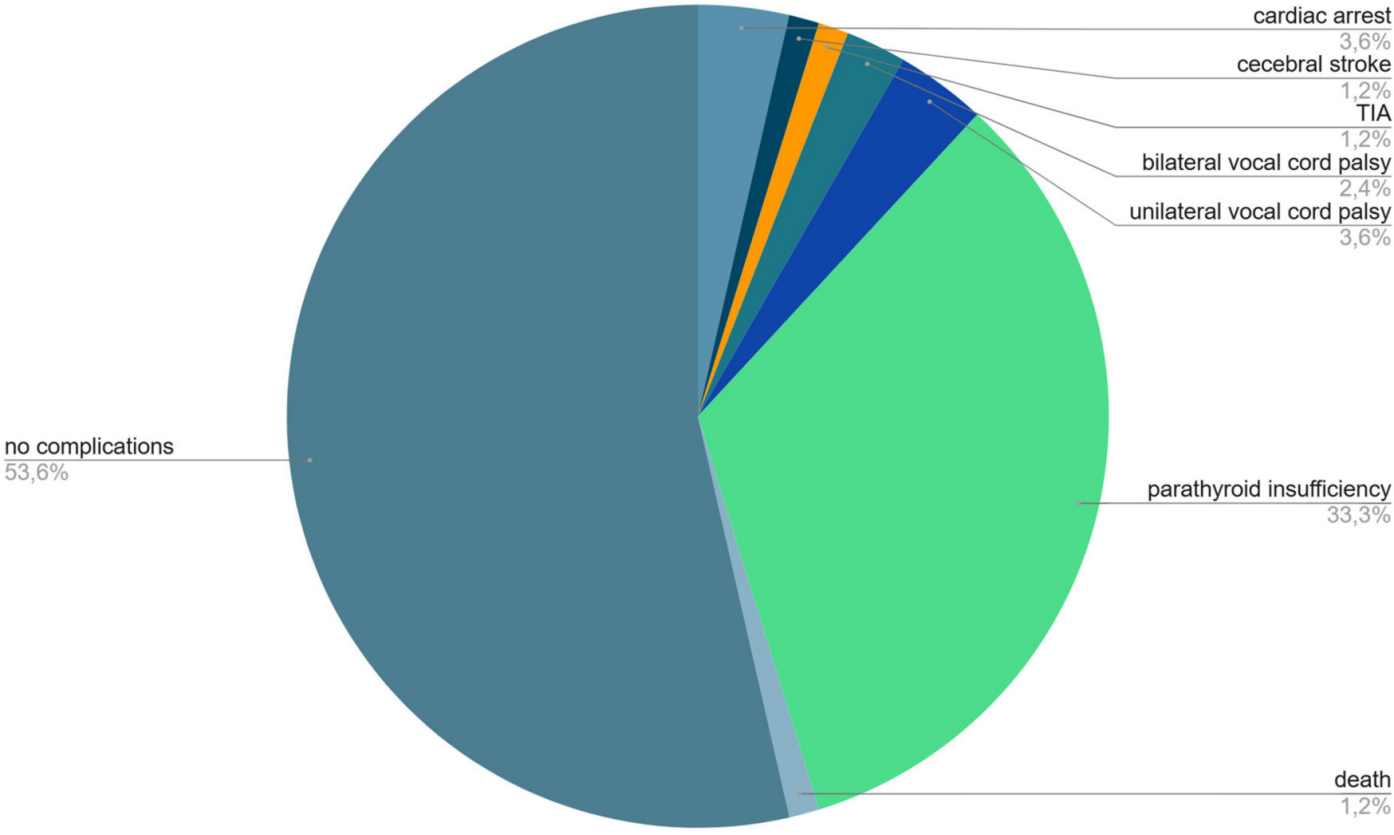
Distribution of complications related to postoperative bleeding and reoperation following thyroid surgery. Over half of the patients (53.6%) had no complications, while parathyroid insufficiency was the most common adverse outcome (33.3%). Less frequent complications included unilateral (3.6%) and bilateral (2.4%) vocal cord palsy, cardiac arrest (3.6%), cerebrovascular events such as stroke (1.2%) and transient ischemic attack (1.2%), and death (1.2%).

Paying attention to possible sources of bleeding is very important to prevent the dreaded complications associated with bleeding and reoperation that are summarized in Figure 3 (31).

In conclusion, we can affirm that this clinical case is relevant as the bleeding occurred at home in a period in which such a complication was unpredictable; fortunately, timely treatment led to a resolution without further consequences, even if bleeding appearing so late has a slower evolution.

Adequate patient education is crucial in the context of early or very early discharge following thyroid surgery. Providing clear, structured postoperative instructions, including guidance on recognizing warning signs such as neck swelling, dyspnea, dysphonia, or symptoms of hypocalcemia, is essential to ensure patient safety after discharge. Emphasis should be placed on the potential timing of late complications, such as hematoma formation or hypoparathyroidism, to facilitate prompt medical intervention when necessary. The use of standardized educational materials, combined with effective communication between the surgical team and the patient, can significantly improve postoperative outcomes and reduce the risk of delayed complication detection.

This case illustrates that postoperative hematoma can, in rare circumstances, occur even beyond the first postoperative week. As prolonged hospitalization for all thyroidectomy patients is neither feasible nor justified, the key lesson is the importance of a patient-centered strategy. Such an approach should include thorough risk stratification, extended postoperative observation (at least 6–8 h in outpatient settings), provision of both verbal and written discharge instructions, and individualized perioperative management of antiplatelet therapy. Empowering patients and their caregivers to promptly recognize and act on warning signs is critical to reducing morbidity and mortality associated with delayed bleeding.

In consideration of all the above reasons, we believe that, although most bleeding occurs within 24 h, caution should be taken in patients on oral antiaggregant treatment and close monitoring should be advised at home after discharge, particularly if the therapy has just restarted.

While the primary surgical challenge in thyroidectomy remains the prevention of bleeding, equal emphasis must be placed on strategies to minimize other procedure-related complications, particularly RLN injury. Identification of the laryngeal nerves can be extremely challenging during emergency procedures, especially in operative fields with clots, as in our case. Intraoperative neuromonitoring (IONM) is increasingly employed to preserve the RLN during thyroid and parathyroid surgery. A meta-analysis of eight randomized trials involving 2,521 patients found no statistically significant difference in RLN injury rates between IONM and visual identification, although a trend toward reduced risk with IONM was noted (32). Similarly, a prospective study in high-risk patients reported no significant reduction in RLN injury but demonstrated shorter operative times and reduced hospital stays, supporting its potential cost-effectiveness (33). Overall, anatomical visualization remains the gold standard, while IONM serves as a valuable adjunct in complex or high-risk procedures.

Although uncommon, late bleeding after thyroidectomy has been described in several reports. Calò et al. (34) reported two cases of bleeding on postoperative day 13 in patients treated with low-molecular-weight heparin. Lang et al. (11) analyzed over 5,900 thyroidectomies and found that approximately one-quarter of hematomas occurred >6 h postoperatively, with a minority presenting after 24 h. Burkey et al. (8) and Godballe et al. (24) also included delayed hematomas in their series, while Campbell et al. (35) confirmed that rare events can occur days after discharge. More recently, pseudoaneurysms of the superior thyroid artery have been described as causes of delayed bleeding (19, 36). In addition, randomized trials and observational studies investigating the perioperative management of aspirin therapy occasionally reported late hematomas as secondary outcomes (29, 30). These reports confirm that delayed postoperative hematoma, though exceptional, is a recognized phenomenon and should be considered in patients undergoing thyroidectomy, particularly those resuming antiplatelet or anticoagulant therapy.

Take-away lesson: Delayed bleeding, although rare, should be considered in thyroidectomy patients resuming antiplatelet therapy. Extended post-discharge monitoring may prevent life-threatening complications.

## Patient perspective

**“**After the surgery, I was feeling well and relieved to return home. When I noticed the swelling and bleeding from the wound several days later, I became very anxious, especially since I had no trouble breathing. I'm grateful for the quick response of the medical team, who explained everything clearly and took immediate action. The second surgery was unexpected, but it went smoothly, and I felt well taken care of. I'm now doing fine and feel reassured knowing the issue was handled promptly. I understand now how important it is to follow up closely, even after you think you're out of the danger zone.”

## Data Availability

The raw data supporting the conclusions of this article will be made available by the authors, without undue reservation.
